# Clinical implications of neoepitope landscapes for adult and pediatric cancers

**DOI:** 10.1186/s13073-017-0470-9

**Published:** 2017-08-31

**Authors:** Yang-Yang Feng, Obi L. Griffith, Malachi Griffith

**Affiliations:** 1 McDonnell Genome Institute, Washington University School of Medicine, St. Louis, MO USA; 2 Division of Oncology, Department of Medicine, Washington University School of Medicine, St. Louis, MO USA; 3 Department of Genetics, Washington University School of Medicine, St. Louis, MO USA; 4 Siteman Cancer Center, Washington University School of Medicine, St. Louis, MO USA

**Keywords:** Exome sequencing, Immunotherapy, Neoepitope, Personalized cancer vaccine, RNA sequencing, Whole-genome sequencing

## Abstract

Many immunotherapies rely on the presence of neoepitopes derived from somatic mutations that lead to altered peptide sequences. Several studies have now analyzed the neoepitope landscape of different cancer subtypes, predominantly for adult samples, which tend to feature significantly higher mutational burden. However, a new report publishing the first comprehensive analysis of the pediatric neoepitope landscape suggests that immunotherapies could also hold promise for pediatric cancers.

See related research article 10.1186/s13073-017-0468-3

## Leveraging the adaptive immune system in cancer treatment

There is now an impressive array of diverse strategies for leveraging the immune system as a promising treatment avenue in several cancer types [1]. A subset of these involve the adaptive immune system, whereby T cells are directed to tumor cells presenting a tumor-specific mutant antigen that is recognized by a receptor on the T cell [2]. The tumor-specific antigen corresponds to any somatic mutation that results in a protein coding difference compared with the wild-type protein sequence. After intracellular processing and cleavage within the tumor cell, each mutant protein sequence can result in one or more distinct peptides of 8–15 amino acids [3]. A subset of these peptides, referred to as neoepitopes, are bound by major histocompatibility complex (MHC) class I or II molecules (encoded by *HLA* genes) and presented on the surface of the tumor cell, where they can be recognized by CD8+ or CD4+ T cells, respectively. T-cell receptor (TCR) sequence diversity resulting from recombination at TCR loci allows for the potential recognition of almost any peptide sequence and, by extension, almost any tumor-specific neoepitope. Upon successful formation of a TCR–MHC–peptide complex, a signal cascade triggers apoptosis and death of the recognized tumor cell. Elucidating each of the major components of this process has been enabled by recent developments in next-generation sequencing. An emerging discipline of ‘immunogenomics’ seeks to systematically characterize the diversity of *HLA* alleles, identify patient-specific HLA haplotypes, identify tumor-specific neoepitopes, predict peptide–MHC binding affinities, match these to specific TCR sequences, and track overall changes in the TCR repertoire [1]. Several relatively recent cancer-treatment modalities have particularly benefited from these advances in immunogenomic profiling and analysis. For example, several studies have used the neoepitope burden to predict which patients might respond to checkpoint-blockade inhibition therapies [4, 5]. In addition, personalized cancer vaccines rely entirely upon identifying a set of promising neoepitopes for each patient [6].

## Neoepitope landscapes as an indicator of immunotherapy potential

In this issue of *Genome Medicine*, Chang and colleagues [7] report the first comprehensive analysis of the neoepitope landscape specifically for pediatric cancers. This landscape joins several others that have focused on pan-cancer analysis of the (mostly) adult tumors represented in The Cancer Genome Atlas (TCGA) project [8] or specific cancer types [9]. From 540 primary tumors, Chang and colleagues identified at least one predicted neoepitope in 78.1, 88.4, and 89.8% of pediatric central nervous system, leukemia, and solid malignancies, respectively. This finding is remarkable and of potential clinical significance owing to the relatively low mutation burden of most pediatric tumors compared with those observed in adults, particularly those associated with prolonged environmental exposures (e.g., smoking for lung cancer and UV for melanoma). Childhood tumors are also often characterized by structural variation; thus, the authors wisely considered RNA expression data, where available, to predict neoepitopes arising from gene fusions in addition to single-nucleotide variants (SNVs). After requiring evidence for RNA expression of each candidate, an estimated 60% of tumors had at least one predicted neoepitope. While many additional factors (several of which are discussed below) remain to be incorporated into neoepitope landscapes for patient cohorts and individual patients, the study provides a promising overview of the potential efficacy of adaptive immune-therapy approaches in pediatric cancer.

## Accurate neoepitope identification for personalized medicine

Chang and colleagues used whole-genome sequencing (WGS) and RNA-seq data to identify two types of somatic variants, specifically SNVs and RNA fusions. For each somatic variant, distinct peptide nonamers were extracted by tiling across the SNV or fusion junction position. *HLA* alleles and nonamers for each patient were used to predict the peptide–MHC binding affinity using a single algorithm. There are several ways in which developing neoepitope-prediction methods could improve such efforts in the future. In addition to the SNVs considered by most studies, insertions and deletions, particularly those resulting in frameshifts, might prove a rich source of neoepitopes [10]. Two of the most comprehensive neoepitope landscape reports to date—Charoentong et al. [8] and Chang et al.—do not appear to consider insertions or deletions in their neoepitope identification, likely because most existing software packages do not yet support neoepitope prediction for indels. However, unlike most previous studies, Chang and colleagues did incorporate neoepitope prediction from gene fusions. A conceptually similar but untapped source of novel peptide epitopes are those created by tumor-associated aberrant RNA splicing events. Furthermore, as different transcript isoforms for the same gene can feature varying reading frames, a single variant might produce multiple unique mutant peptides. Therefore, understanding the alternative splicing profile of a tumor will be imperative for identifying which neoepitopes are actually expressed. Many additional factors remain unexplored that could prove useful in prioritizing neoepitopes for use in personalized cancer vaccines or predicting response to immunotherapy. For example, the importance of varying the peptide length or the mutation position within the peptide sequence remains unclear. In addition, cross-reactivity of neoepitopes with wild-type peptides and how this affects self/non-self determination by the immune system is poorly understood. The minimum or optimal number of peptides required for clinical response also remains unknown.

Each of these example factors presents an opportunity to improve the process of neoepitope landscape characterization. Over the past few years, we have moved from simple mutation burden as a predictor of possible response to immunotherapy, to neoepitope burden, and now expressed neoepitope burden. It seems likely that not all neoepitopes with MHC binding affinity below an arbitrary threshold (e.g., IC50 < 500 nm) are created equal. A weighted neoepitope score that incorporates additional predictive features could lead to more clinically relevant neoepitope landscapes. Crucial to the development of such a score will be the publication of large datasets of experimentally and clinically validated neoepitopes.

## Potential for clinical impact of neoepitopes in pediatric and adult cancers

**Fig. 1:**
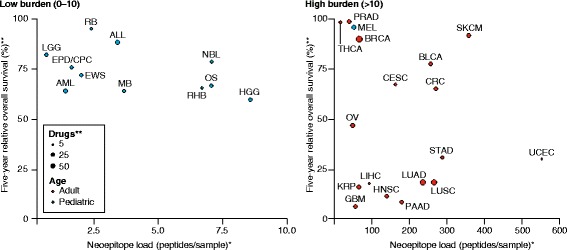
Five-year relative survival versus neoepitope load in pediatric and adult cancers. Five-year relative survival is plotted against average neoepitope load for 29 cancer subtypes. *Dot size* represents the number of therapies approved by the US Food and Drug Administration (range 1 to 67) that are indicated for each specific disease. Survival data were obtained from the SEER Cancer Statistics Database (https://seer.cancer.gov), and drug counts for each cancer type were obtained from the A to Z List of Cancer Drugs provided by the National Cancer Institute (https://www.cancer.gov). Neoepitope load numbers are derived from the analyses presented in Chang et al. [7] and Charoentong et al. [8] for children (*blue*) and adults (*red*), respectively. *Neoepitope loads represent peptide–HLA binding predictions for missense single-nucleotide variants (SNVs) only. **Drug counts and survival rates for certain displayed subtypes are approximations as the NCI and SEER have grouped cancers differently compared with the sources of neoepitope load data. *Abbreviations*: *ALL* acute lymphoblastic leukemia, *AML* acute myeloid leukemia, *BLCA* bladder urothelial carcinoma, *BRCA* breast invasive carcinoma, *CESC* cervical squamous cell carcinoma and endocervical adenocarcinoma, *CRC* colon-rectum adenocarcinoma, *EPD*/*CPC* ependymomas and choroid plexus tumor, *EWS* Ewing sarcoma, *GBM* glioblastoma multiforme, *HGG* high-grade glioma, *HNSC* head and neck squamous cell carcinoma, *KRP* kidney and renal pelvis tumors, *LGG* low-grade glioma, *LIHC* liver hepatocellular carcinoma, *LUAD* lung adenocarcinoma, *LUSC* lung squamous cell carcinoma, *MB* medulloblastoma, *MEL* melanoma, *NBL* neuroblastoma, *OS* osteosarcoma, *OV* ovarian serous cystadenocarcinoma, *PAAD* pancreatic adenocarcinoma, *PRAD* prostate adenocarcinoma, *RB* retinoblastoma, *RHB* rhabdomyosarcoma, *SKCM* skin cutaneous melanoma, *STAD* stomach adenocarcinoma, *THCA* thyroid carcinoma, *UCEC* uterine corpus endometrial carcinoma

It is notable that such a high proportion of the pediatric cases in Chang et al.’s study is identified as having at least one potential neoepitope. When the neoepitope landscape is considered in the context of 5-year relative survival and availability of approved drugs, several pediatric and adult cancer types appear particularly promising for response to immunotherapy. For example, pediatric high-grade glioma (HGG), adult uterine corpus endometrial cancer (UCEC), and several others are characterized by relatively high neoepitope burden, low survival rates and relatively few approved therapies (Fig. 1). Melanoma (MEL) has shown promising clinical responses in adult immunotherapy and features an exceptionally high neoepitope load, even in pediatric cases.

Given the understandable limitations of the analyses completed to date, the potential for application of immunotherapies in pediatric cancer could be even more promising than it currently seems. Owing to the complexities discussed above, it is likely that the existing neoepitope landscapes underestimate the number of potential tumor-specific mutant antigens. However, there is also reason for tempered optimism. The undeniably low neoepitope burden in most pediatric and some adult tumors poses a challenge in leveraging a specific immune response. Furthermore, reports of transient treatment response followed by progressive disease necessitate further investigation into development of resistance to these emerging immunotherapies. Nevertheless, there is reason to hope that certain cancers with high neoepitope burden and upregulated checkpoint blockade pathways will respond to either personalized vaccines or checkpoint blockade inhibition. The combination of these two therapies could be effective in an even larger proportion of the patient population. Studies like that of Chang and colleagues suggest that improving our understanding of the neoepitope landscape of each tumor type will be a key component of identifying these patients.

## Abbreviations

HLA: Human leukocyte antigen
MHC: Major histocompatibility complex
SNV: Single-nucleotide variant
TCR: T-cell receptor
